# Control of fly strike dermatitis in dogs with a topically applied combination of imidacloprid and permethrin: a prospective open-label controlled clinical trial

**DOI:** 10.1186/s13071-019-3356-4

**Published:** 2019-03-21

**Authors:** Eloy Castilla-Castaño, Fabien Moog, Caroline Mandin-Cabaret, Charline Pressanti, Marie Christine Cadiergues

**Affiliations:** 1 Université de Toulouse, ENVT, Small Animal Hospital, Dermatology Service, 23, Chemin des Capelles, 31076 Toulouse cedex3, France; 2 Clinique Vétérinaire, 1 Avenue Léon Blum, 31500 Toulouse, France; 3 UDEAR, Université de Toulouse, INSERM, ENVT, 23, Chemin des Capelles, 31076 Toulouse Cedex3, France

**Keywords:** Dogs, Dermatology, Muscidae, *Stomoxys calcitrans*, Antiparasitic agents, Insecticides, Therapeutics

## Abstract

**Background:**

A prospective clinical study evaluated the tolerance and the efficacy of a combination of imidacloprid (10%) and permethrin (50%) (ADVANTIX^®^: Bayer HC AH, France) applied topically as a spot-on, for the treatment of natural canine fly dermatitis due to *Stomxys calcitrans*. The study was an open-label controlled study and one-month follow-up.

**Methods:**

Fifteen dogs, from the same animal kennel, with active pinnal lesions of fly dermatitis, received a single application of the solution on the cranium and the base of the ears on Day 0 (D0). Five dogs, from the same kennel, similarly affected, served as non-treated controls. No other therapeutical or hygienic measures were taken. Lesional score was based on extension, alopecia, crusts, scales, erosions/ulcers, loss of substance and lichenification, each assessed on a 0–4 scale. Evaluation was performed on D0, D14 and D30. Total lesion score reduction was calculated at each time point using the arithmetic mean of total lesion score according to Abbott’s formula. Scores obtained on D14 and D30 were compared with the baseline obtained on D0.

**Results:**

No adverse event was recorded. The lesion score ranged between 4–13 at D0 in all dogs. In control dogs, D0 mean (± SD) lesion score was 7 ± 1.4. Lesion scores were maintained on D14 (6.6 ± 3.4) and D30 (8.6 ± 5.4). In treated dogs, D0 mean lesion score was 9.9 ± 2.5. Lesion scores of the treated dogs were reduced by 59% on D14 (4.1 ± 2.8) and 80% on D30 (1.9 ± 1.5) (*P* < 0.05).

**Conclusions:**

The combination imidacloprid-permethrin proved safe and helpful in the management of natural canine fly dermatitis. It could also be suggested as a preventive measure with a monthly application during the fly exposition phase.

## Background

Within the order Diptera, along with families such as the Ceratopogonidae and Simulidae, flies of the family Muscidae play an important pathogenic role, both direct (bites, fly-worry) and indirect (vectors of bacterial, helminth and protozoal diseases), in livestock [1] and people [2] but also in pets [3]. In dogs, the stable fly, *Stomoxys calcitrans* Linné, 1758, is associated with the so-called “fly strike dermatitis”. Stable flies bite dogs preferentially on the ear flap, sucking blood. In dogs with erected ears, lesions are typically seen on the tip of the flap [3, 4], whereas in dogs with pendulous ears, lesions are present on the folding edge [4]. Both sexes are haematophagous and bite usually twice daily [3]. Fly strike dermatitis requires a continuous exposure to stable flies and lesions tend to disappear in the absence of bites. Dogs living outdoors especially confined and close to livestock, are more prone to developing lesions. The clinical expression is seasonal, peaking in summer and fall. Typical lesions are erythema and dark crusty material secondary to post-bite serum and blood oozing. Alopecia and ulcerations are possible, usually secondary to pruritus, which is not always present [4].

There are limited published data on the benefit of insecticidal products against the stable fly in dogs and few products are specifically licensed for this purpose. Published reports are limited to controlled studies of experimental infestations, with permethrin combined with imidacloprid [5] or fipronil [6]. The repellent properties of permethrin have been evaluated as having an anti-feeding effect (prevention of blood meal); the insecticidal efficacy (killing effect) has also been assessed [5, 6]. No report is available concerning clinical efficacy against dermatitis caused by flies.

The purpose of the present study was to provide additional field efficacy data to complete results obtained under experimental conditions [5]. It aimed to evaluate the ability of the repellent product to indirectly treat fly strike dermatitis in clinically affected dogs by limiting the incidence of the fly bites with a combination 10% imidacloprid and 50% permethrin spot-on solution (Advantix^®^; Bayer HC AH, France) applied topically as a spot-on in dogs with natural exposition to *S. calcitrans* in south-west France (Toulouse). This study was designed as a single treatment, open-controlled study.

## Results

**Fig. 1:**
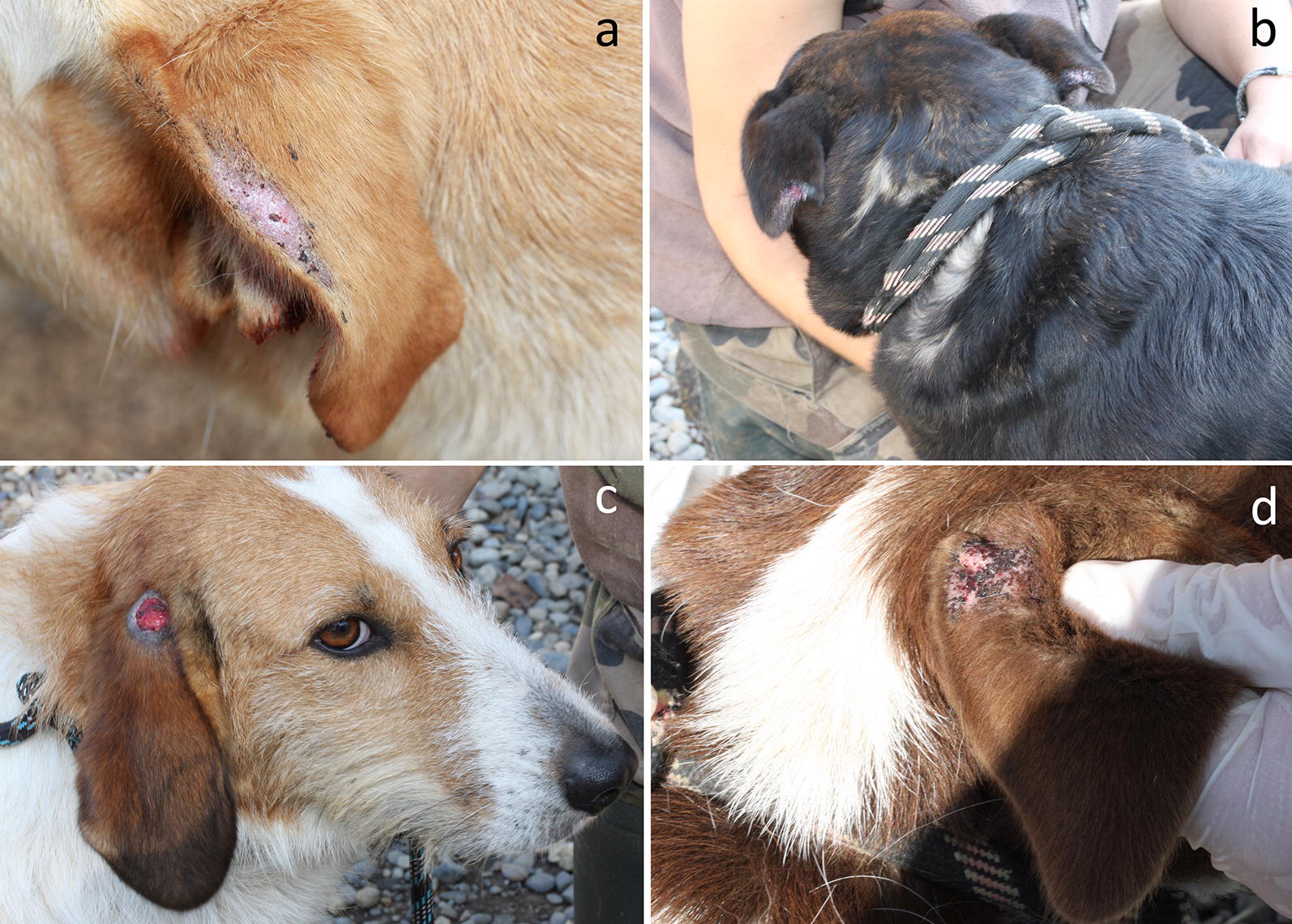
Distribution of skin lesions on the ear pinna in dogs with semi-erected ears (**a**, **b**) and pendulous ears (**c**, **d**) affected by fly-strike dermatitis

Of the 20 dogs initially included, 19 completed the study. One dog was adopted before the second visit and was therefore excluded. There were 12 females (2 control and 10 treated dogs) and 7 males (3 control and 4 treated dogs) aged between 1 and 10 years (median = 5) and weighing between 18 and 55 kg. All were mongrel dogs, mostly Labradors (6), Griffons (4) Shepherds (4) and cross-breeds. All had floppy (8) or semi-erected (11) ears. Lesions were observed on the anterior (Fig. 1a) and/or posterior (Fig. 1b) margins of the pinnae of dogs with semi-erected ears, whereas they were noticed on the base of the pinna on dogs with floppy ears (Fig. 1c, d; Table 1). No local or systemic adverse effect was observed in any dog during the study.

**Table 1:** Characteristics of the animals and clinical features at D0 and D30

Animal no.	Breed	Sex	Age (years)	Weight (kg)	Type of ears	Clinical score at D0	Clinical score at D30	Location of the lesions on the pinna	Dominant type of lesion	Treatment received at D0
1	Griffon	M	2	20	Flp	13	2	Base	Ulcer	Advantix^®^
2	Shepherd cross	M	1	18	SE	11	2	Anterior margin	Alopecia, black crusts	Advantix^®^
3	Griffon cross	M	1	22	Flp	12	3	Base	Ulcer	Advantix^®^
4	Shepherd cross	M	1.5	25	SE	5	3	Anterior margin	Alopecia	None
5	Rottweiler cross	F	7	55	SE	4	0	Anterior margin	Alopecia	Advantix^®^
6	Labrador retriever cross	F	3	30	SE	7	2	Anterior margin	Ulcer, alopecia	Advantix^®^
7	Griffon cross	F	2	19	Flp	10	3	Base	Ulcer	Advantix^®^
8	Shepherd cross	F	2	24	SE	9	2	Anterior and posterior margins	Alopecia, black crusts	Advantix^®^
9	Labrador retriever	F	9	50	Flp	11	4	Posterior margin	Ulcer	Advantix^®^
10	Labrador retriever cross	F	4	30	Flp	8	10	Base	Alopecia	None
11	Rottweiler crosed beauceron	M	6	48	Flp	10	5	Base	Ulcer	Advantix^®^
12	Briard cross	F	10	40	Flp	12	2	Base	Ulcer, black crusts	Advantix^®^
13	Griffon cross	M	4	45	SE	6	3	Posterior margin	Alopecia	None
14	Staffordshire terrier cross	F	6	18	SE	8	0	Anterior margin	Alopecia	Advantix^®^
15	Shepherd cross	F	8	41	SE	12		Anterior margin	Ulcer	Excluded
16	Labrador retriever cross	M	10	45	SE	8	15	Anterior margin	Alopecia, black crusts	None
17	Border collie cross	F	5	22	Flp	10	2	Base	Ulcer	Advantix^®^
18	Labrador retriever cross	F	5	43	SE	12	0	Posterior margin	Ulcer, black crusts	Advantix^®^
19	Labrador retriever cross	F	7	45	SE	7	0	Anterior and posterior margins	Alopecia, black crusts	Advantix^®^
20	Hunting breed cross	F	5	20	SE	8	12	Posterior margin	Alopecia, black crusts	None

*Abbreviations*: M, male; F, female; SE, semi-erected ears; Flp, floppy ears

**Fig. 2:**
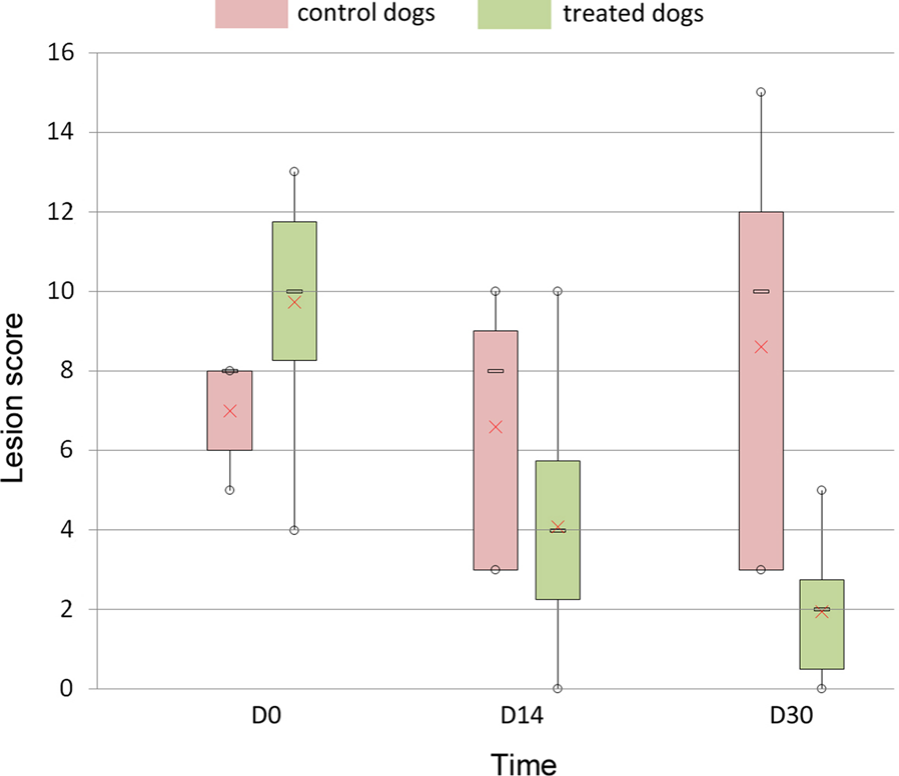
Boxplot of the progression of the lesion score of control dogs (red) and treated dogs (green) affected by fly strike dermatitis at D0, D14 and D30 after a single application of a topical combination of imidacloprid (10%) and permethrin (50%) (Advantix^®^, Bayer HC AH, France). A significant and continuous reduction in the lesion score is observed in the treated dog group compared to the control group. The horizontal line within the box indicates the median, boundaries of the box indicate the 25th and 75th percentile, and the whiskers indicate the highest and lowest values of the results. The “×” marked in the box indicates the mean

The lesion score ranged from 4 to 13 (maximal possible score 28) at Day (D)0 in all dogs. In control dogs, D0 mean (± SD) lesion score was 7 ± 1.4. Lesion scores were maintained at D14 (6.6 ± 3.4) and D30 (8.6 ± 5.4), which confirmed the continuous presence of biting flies and adequate parasitic pressure, as natural progression in the absence of flies would be improvement up to clinical cure. In treated dogs, D0 mean lesion score was 9.9 ± 2.5. Lesion scores of the treated dogs were reduced by 59% on D14 (4.1 ± 2.8) and 80% on D30 (1.9 ± 1.5), (Fig. 2). The data analysis revealed a significant difference in reduction of lesion score between D0 and D14 (Wilcoxon signed-rank test: *Z* = -3.2958, *P* < 0.001) and between D0 and D30 (Wilcoxon signed-rank test: *Z* = -3.2958, *P* < 0.001), in treated dogs, respectively.

## Discussion

Stable flies (*Stomoxys calcitrans*) cause irritation and pain at the site of the bite and repeated attacks can lead to open wounds. It is considered to be a serious nuisance in exposed animals, causing continuous irritation and restlessness in the daylight hours [5]. Skin damage caused by the repeated insertion of the rigid proboscis results in oozing of blood and serum and in severe cases significant ulceration and necrosis. The lesions and their distribution which were observed on D0 were consistent with the literature reports [3–5], although insect traps were not used to confirm the presence of *S. calcitrans* in the kennel. Nevertheless, the presence of *S. calcitrans* in the geographical area has already been documented [7]. In addition to possible etiologic factors which were excluded, leishmaniosis was considered unlikely based on the absence of general symptoms and other dermatologic abnormalities and the rapid onset of the lesions over the past two months on a large numbers of dogs of the sanctuary. However, serologic tests were not done. This study evaluated the efficacy of a topical combination of permethrin and imidacloprid to control skin lesions of canine fly dermatitis in exposed kennel dogs. Assessment of efficacy was based on variation of lesion scoring of affected dogs. Pinnal lesions are due to repeated stable fly bites. By preventing or decreasing the numbers of bites, skin is no longer or less aggressed and lesions can spontaneously improve.

We elected to include a control group to ensure that the reduction of the lesions in dogs receiving the treatment was not spontaneous. As fly strike dermatitis is (i) a seasonal parasitic disease and (ii) requires a continuous exposure to the stable flies and that lesions tend to disappear in the absence of bites, in the absence of a control group, the improvement of the lesions on treated animals could be misinterpreted. In addition to live in runs close to those of treated dogs, we based the selection of control dogs on lesions similar to that of treated dogs. However, we found it more ethical to only select control dogs with lesions of lower severity as they would remain untreated. Lesion score of dogs from the control group tended to increase. Control dogs received a rescue treatment on D30 with the same product.

The label of the permethrin-imidacloprid spot-on formulation recommends a topical application in four spots on the back from the shoulder to the base of the tail. This formulation allows the product to spread on the skin from the application spot and the concentration is expected to be minimal at the extremities, the most distanced body regions [8] including ears. We therefore purposely followed an off-label application of the product: near to the ear flaps (along each ear base and on the cranium) to reach a higher concentration of the product where the bites usually occur.

The significant and continuous decrease in lesion scores from D0 to D30 in the absence of hygienic measures and other forms of treatment confirmed that the repellent effect of the product was sufficient to allow healing of the lesions by preventing blood-feeding. At D30, the lesion score of treated dogs was reduced by 80% compared to baseline. No cutaneous or systemic adverse effect was noted in the treated group.

These results provide additional information to the previous studies that evaluated the repellent effects of pyrethroids on arthropods [9] associated with imidacloprid in laboratory and outdoor conditions [10–13].

The efficacy of the formulation combining permethrin (50%) and imidacloprid (10%) against *Stomoxys calcitrans* was evaluated under laboratory conditions [5]. The data obtained under field conditions confirm the good repellent efficiency of the product.

## Conclusions

The results of the present study demonstrate that a single topical treatment of the combination of imidacloprid (10%) and permethrin (50%) is safe and helpful in the management of natural canine fly dermatitis in a kennel environment. It confirms previous results in preventing *Stomoxys calcitrans* from taking a blood meal on experimentally infested dogs during a period of 29 days after a single application.

The results also suggest that in endemic areas, it could be used as a preventive measure against fly dermatitis with a monthly application during the fly exposition phase.

## Methods

A total of 20 dogs sheltered in an animal sanctuary were recruited in the south-west of France. Both sanctuary manager written consent and approval from the Toulouse veterinary school (Université de Toulouse, ENVT) Ethical Committee were obtained prior to beginning the study. Fly-strike dermatitis was diagnosed based on epidemiology (end of summertime, confined dogs housed permanently outdoors, in close proximity to donkeys and horses and presence of numerous flies), clinical signs (erythema, hemorrhagic crusts and erosions/ulcerations on the tips of the ears of dogs with erected pinnae or at the folded edge of the skin in dogs with pendulous ears) and exclusion of other etiologic factors (sarcoptic mange, otodectic mange, harvest mites infestation, secondary microbial pinnal and aural infections) by appropriate tests (skin scrapes, lesion surface and ear canal cytologies).

Inclusion criteria were the presence of pinnal lesions caused by flies on D0 and the absence of change in the geographical location in the sanctuary of the dog for the duration of the study.

Fly strike dermatitis is a seasonal skin disease. The study was conducted during October and depending on the year, in the study area, the first night frosts in the autumn can occur by the end of September. As a consequence, the fly population could decrease. Therefore five dogs were included in parallel and left untreated serving as sentinels, to ensure that the tested population would be exposed to potential fly bites throughout the study. Exclusion criteria included dogs with systemic illness or condition which could deteriorate within the following month, and dogs who could be adopted before the end of the study. Dogs having received an insecticidal treatment within the past four weeks were also excluded.

The study consisted of three visits. Dermatological evaluations were conducted on the day of inclusion (D0), D14 and D30 or closing visit. Each case was evaluated by the same investigator throughout the study. Lesion score was based on (i) 6 possible different types of lesions: [alopecia, crusts, erosions/ulcers, scales, loss of substance and lichenification; each lesion type was scored independently on a 0–4 scale (0, none; 1, very mild; 2, mild; 3, moderate; 4, severe)] and (ii) extension of the lesions (0, absence of lesion; 1, only one ear flap and over a width less than 2 mm; 2, both ear flaps over a width between 2–9 mm; 3, both ear flaps over a width between 10 mm and the 2/3 of the surface; 4, both ear flaps on more than 2/3 of their surface). Scores were recorded for each dog at each time point for each lesion and the total lesion score was calculated for each dog as the sum of each lesion score and extent score. The maximum score was 28.

On study D0, each dog in the treated group received a topical application of the combination of permethrin/imidaclopride (Advantix^®^, Bayer HC AH, France) at the commercial dose of the product based on bodyweight (pipette dose). The product was applied off-label. The tip of the tube was gently placed on the dog’s skin after having parted the fur with the fingers. About 1/3 of the pipette was applied along each ear base and 1/3 was applied on the cranium. Lesion areas were avoided. No other topical or systemic medications were allowed during the study duration. The variable was the resolution of clinical signs. Total lesion score reduction was calculated at each time point (t) using the arithmetic mean of total lesion score according to Abbott’s formula: Total lesion score reduction (%) = 100 × (mean D0 − mean day t)/mean D0.

Wilcoxon signed-rank tests were used to compare scores obtained on D14 and D30 with the baseline obtained on D0. Significance was defined as *P* < 0.05. All statistical analysis was performed using XLSTAT 2017-02 (Addinsoft SARL, Paris, France).
